# Storage cell proliferation during somatic growth establishes that tardigrades are not eutelic organisms

**DOI:** 10.1242/bio.060299

**Published:** 2024-02-27

**Authors:** Gonzalo Quiroga-Artigas, María Moriel-Carretero

**Affiliations:** Centre de Recherche en Biologie cellulaire de Montpellier (CRBM), Université de Montpellier, Centre National de la Recherche Scientifique, 34293 Montpellier CEDEX 05, France

**Keywords:** Tardigrade, Storage cell, Eutely, EdU, Cell proliferation, DNA replication

## Abstract

Tardigrades, microscopic ecdysozoans known for extreme environment resilience, were traditionally believed to maintain a constant cell number after completing embryonic development, a phenomenon termed eutely. However, sporadic reports of dividing cells have raised questions about this assumption. In this study, we explored tardigrade post-embryonic cell proliferation using the model species *Hypsibius exemplaris*. Comparing hatchlings to adults, we observed an increase in the number of storage cells, responsible for nutrient storage. We monitored cell proliferation via 5-ethynyl-2′-deoxyuridine (EdU) incorporation, revealing large numbers of EdU^+^ storage cells during growth, which starvation halted. EdU incorporation associated with molting, a vital post-embryonic development process involving cuticle renewal for further growth. Notably, DNA replication inhibition strongly reduced EdU^+^ cell numbers and caused molting-related fatalities. Our study is the first to demonstrate using molecular approaches that storage cells actively proliferate during tardigrade post-embryonic development, providing a comprehensive insight into replication events throughout their somatic growth. Additionally, our data underscore the significance of proper DNA replication in tardigrade molting and survival. This work definitely establishes that tardigrades are not eutelic, and offers insights into cell cycle regulation, replication stress, and DNA damage management in these remarkable creatures as genetic manipulation techniques emerge within the field.

## Teaser

**Summary:** This study demonstrates active storage cell proliferation in tardigrade post-embryonic development. Storage cells replicate in bursts during molting, especially in juveniles, and DNA replication disruption induces molting-related fatalities.

## INTRODUCTION

**Fig. 1.**
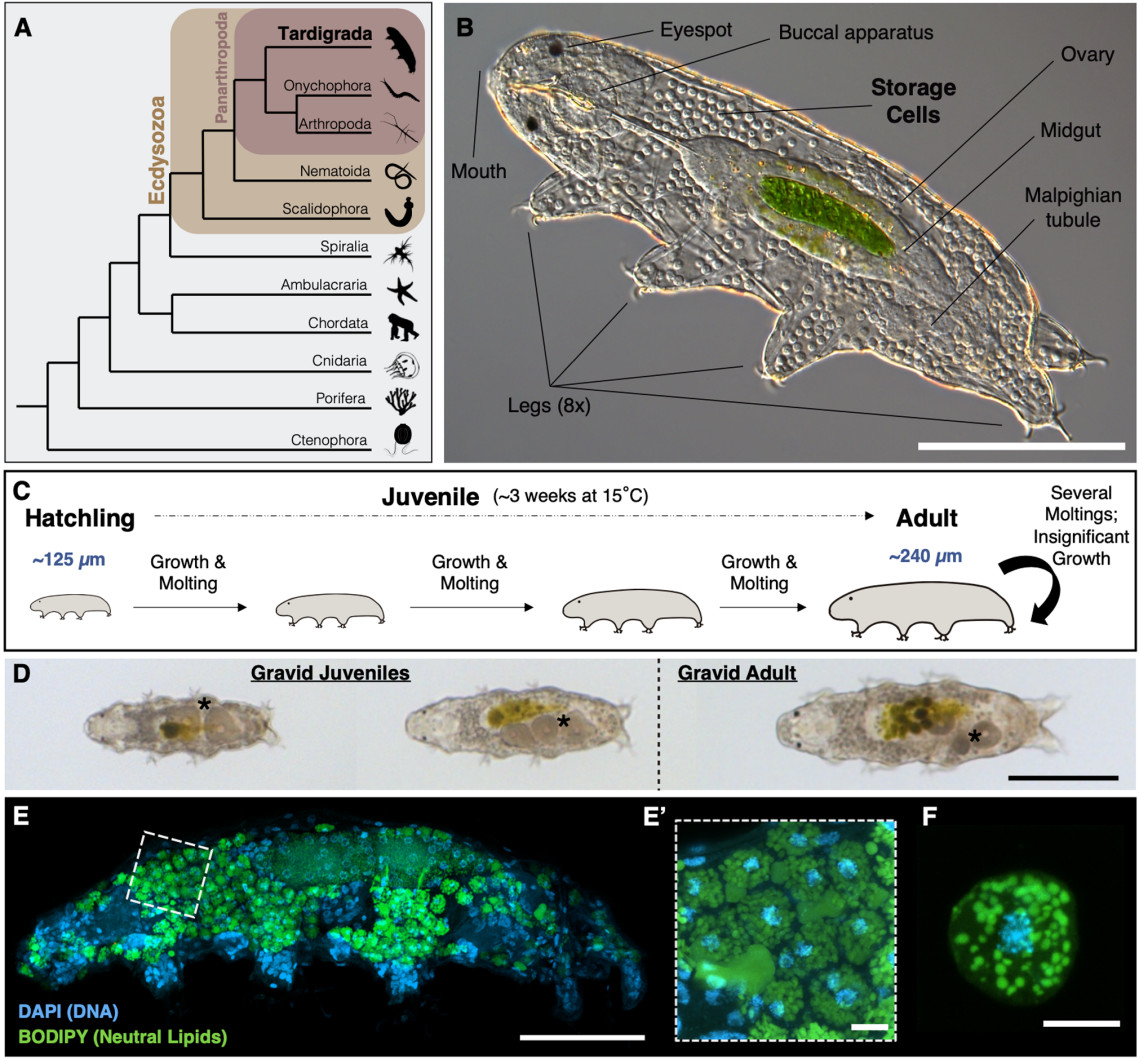
**The tardigrade *Hypsibius exemplaris* and its storage cells.** (A) A simplified animal phylogeny, highlighting the position of tardigrades (Tardigrada) within the Ecdysozoa. Tardigrades share common ancestry with the Onychophora and Arthropoda group, collectively forming the Panarthropoda clade (after Schultz et al., 2023; Wu et al., 2023; Laumer et al., 2019; Dunn et al., 2014; and Campbell et al., 2011). Certain groups like Placozoa and Xenacoelomorpha are omitted for simplicity. Images sourced from Phylopic.org. (B) Anatomy of *H. exemplaris*. Internal organs are revealed in a smushed specimen. The cuticle refracts light and envelops the animal, while the green patch inside the midgut indicates the digestion of *Chlorococcum* algae. (C) Schematic representation of *H. exemplaris* post-embryonic development. (D) Images of two gravid juveniles at different sizes and a gravid fully-grown adult. Black asterisks indicate the location of oocytes. (E) Representative image of a gravid juvenile showing cell nuclei (DAPI, blue) and neutral lipids (BODIPY, green), primarily visible in lipid-rich storage cells. (E′) Magnification of the region outlined in E, emphasizing lipid-filled storage cells. (F) One storage cell isolated from the animal, stained as in E. The full depth of the tardigrade (E) and of the storage cell (F) was captured in confocal z-stacks, and the images shown are maximum projections. In E′, a sub-stack was used to facilitate lipid droplet visualization in the storage cells' cytoplasm. Scale bars: 100 µm in B, D; 50 µm in E; 5 µm in E′-F.

Tardigrades, often called water bears, are tiny animals (0.1-1 mm long) that belong to the superphylum Ecdysozoa (Fig. 1A). This clade is characterized by the presence of an exoskeleton or cuticle, which acts as a protection for their bodies (Hickman et al., 2020). Tardigrades' cuticle plays pivotal roles in their life cycle, enabling them to respond and adapt to diverse environmental challenges (Czerneková and Vinopal, 2021). Like other ecdysozoans, tardigrades must undergo a process called ecdysis (i.e. molting), which involves producing a new cuticle and shedding the old one (known as exuvium), in order to grow in size (Czerneková and Vinopal, 2021; Hickman et al., 2020). Tardigrades are renowned for their exceptional capacity to withstand extreme conditions, leading to a significant surge in research on this subject in recent years (Kasianchuk et al., 2023; Møbjerg and Neves, 2021). However, a more comprehensive understanding of tardigrade biology under physiological conditions is also crucial, as it is necessary to really understand the changes they undergo when exposed to harsh scenarios.

Among tardigrades, *Hypsibius exemplaris* (Fig. 1B) is established as an emerging model organism in evolutionary developmental biology and extreme tolerance research (Goldstein, 2022; Goldstein and King, 2016). Like many other tardigrades, it exhibits parthenogenesis and synchronizes its molting period with oviposition to provide additional physical protection to the developing embryos through the exuvium (Goldstein, 2022; Altiero et al., 2018). *H. exemplaris* hatch from their eggshells upon completion of embryonic development, measuring about 125 µm in length, and resembling small adults. They progressively increase in size through multiple molts, eventually reaching a fully-grown adult state measuring approximately 240 µm in length (Yoshida et al., 2019; this work). Subsequently, they continue to molt at a relatively constant rate until their death, although these subsequent molting events no longer contribute to additional growth (Fig. 1C). *H. exemplaris* start producing eggs (i.e. reach sexual maturity) shortly after hatching, long before reaching their fully-grown size (Fig. 1D; Altiero et al., 2018; Vasanthan and Stone, 2020). To simplify the terminology, we named ‘adults’ those tardigrades that had both reached sexual maturity and their fully-grown size, and ‘juveniles’ those that were still growing, irrespective of sexual maturity (Fig. 1C,D).

Tardigrades feature a particular cell type, the storage cells, found floating freely throughout their body cavity fluid (Gross et al., 2019; Hyra et al., 2016; Fig. 1B). Their primary function is to assist in tardigrade nutritional maintenance by storing and distributing energy in the form of protein, glycogen, and fat (Hyra et al., 2016; Reuner et al., 2010). They also participate in vitellogenesis (Hyra et al., 2016; Poprawa, 2006), and potentially contribute to immunity (Volkmann and Greven, 1993). A recent study has shown increased expression of genes belonging to the *SAHS* (secretory abundant heat-soluble) family in storage cells (Tanaka et al., 2023), hinting at their potential involvement in desiccation tolerance. In *H. exemplaris*, lipids constitute the primary reservoir material within these cells (Hyra et al., 2016). These lipids can be stained with vital dyes such as BODIPY (Czerneková et al., 2017; Hyra et al., 2016; see the Materials and Methods), revealing abundant fat in the form of lipid droplets inside the cytoplasm of storage cells (Fig. 1E,F). Storage cells can be isolated from the organism through dissection, enabling independent experimentation with them, separate from the whole organism (Neumann et al., 2009; Fig. 1F).

Eutely refers to a biological phenomenon observed in certain organisms where somatic cell division ceases once embryonic development is complete, implying a relative constancy in cell numbers throughout an animal's growth (Van Cleave, 1932). This suggests that subsequent growth involves an increase in cell size rather than an increase in cell count. Different lines of evidence have indicated that cell numbers in tardigrades are not constant throughout their post-embryonic development. Over 50 years ago, variations in organ cell numbers and potential somatic mitoses among different tardigrade species were already documented (Bertolani, 1970a,b). More recently, two studies pointed at cell proliferation in two different somatic cell types. One study captured a limited number of storage cells presenting condensed chromosomes using DNA staining approaches in the tardigrade *Richtersius coronifer* (Czernekova and Jönsson, 2016). Another study, using *H. exemplaris* under starved conditions, showed molecular evidence of cell proliferation at the anterior and posterior ends of the midgut, a process involved in replacing the cells lining the gut rather than in increasing the overall cell numbers (Gross et al., 2018). Despite these findings, a recurrent narrative found both in peer-reviewed scientific literature (Beltrán-Pardo et al., 2015; Hickman et al., 2020; Kaczmarek, 2021; Milo and Phillips, 2015; Vasanthan and Stone, 2020) and in tardigrade biology outreach websites, portrays tardigrades as eutelic animals. To definitively establish tardigrades as non-eutelic organisms, and to avoid further literature confusion, a more precise characterization of cell proliferation during somatic growth under normal physiological conditions within this enigmatic phylum is needed.

In this study, we used *H. exemplaris* to assess cell proliferation during tardigrade post-embryonic development. Our findings reveal that overall cell numbers increase when comparing hatchlings to adults, mainly attributed to a prominent rise in storage cell numbers. Notably, EdU, a molecular tool for monitoring DNA replication, incorporates into storage cells at significantly higher rates during tardigrade growth than in fully-grown adults. We also show that starvation arrests growth and blocks EdU incorporation in storage cells, altogether linking storage cell proliferation to somatic growth. Moreover, we found a direct association between storage cell DNA replication and ecdysis and reveal that hampering DNA replication provokes animal death during molting. Our results offer a comprehensive insight into DNA replication patterns during tardigrade growth, decisively demonstrating that tardigrades cannot be categorized as eutelic animals.

## RESULTS

### Tardigrade growth is accompanied by an increase in storage cell number

**Fig. 2.**
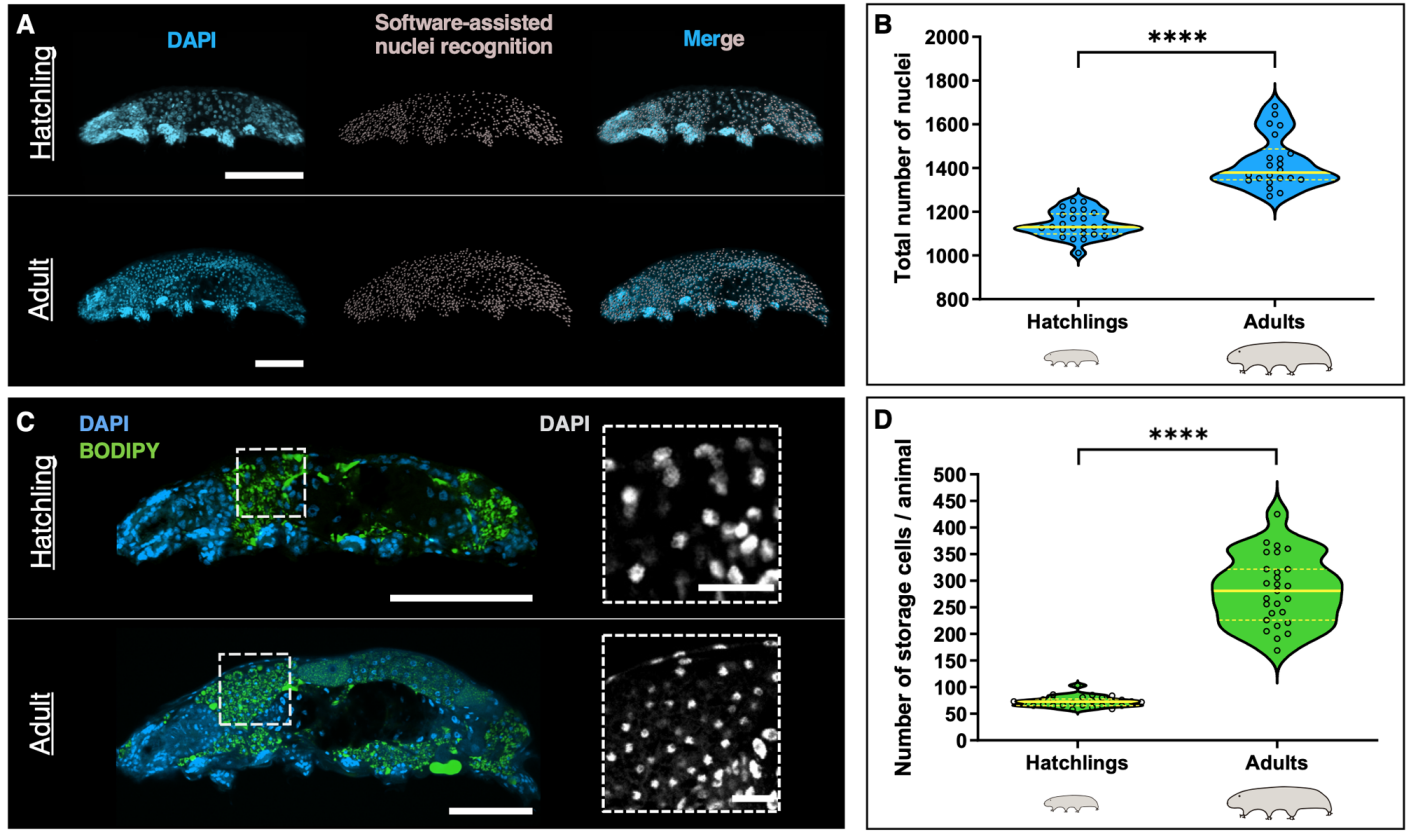
**Somatic growth involves overall cell number increase, mainly driven by storage cell number expansion.** (A) Representative images of a hatchling (top) and an adult (bottom) of *H. exemplaris*, showing nuclei (DAPI, blue) and software-assisted nuclei recognition (in gray; see the Materials and Methods). Rightmost panels show merged images. The full depth of the animals was captured in confocal z-stacks, and the images shown are maximum projections. (B) Violin plots displaying the total number of nuclei in hatchlings (*n*=25) and adults (*n*=22). Center yellow lines show the medians; discontinuous yellow lines show the quartiles; the width of the violin at any given point represents the density of data points at that value; each quantified sample is represented by an empty circle. (C) Representative images of a hatchling (top) and an adult (bottom) of *H. exemplaris*, showing DNA (DAPI, blue) and neutral lipids (BODIPY, green). Insets show magnifications of the regions outlined in C, corresponding to the antero-dorsal region of the tardigrade body, where a big proportion of storage cells are normally found (Fig. 1E; Gross et al., 2019). Nuclei in insets are shown in gray. Individual images are shown, rather than maximum projections, to facilitate storage cell visualization within the animals. (D) Violin plots depicting the number of storage cells in hatchlings (*n*=26) and adults (*n*=27). Violin plot description as in B. ****, *P*≤0.0001. Scale bars: 50 µm in A, C; 10 µm in C insets.

We first collected and fixed animals at both extremes of body size: hatchlings and fully-grown adults (Fig. 1C). We utilized the fluorescent DNA marker DAPI (4′,6-diamidino-2-phenylindole) to quantify the number of cell nuclei present at each growth stage (see the Materials and Methods; Fig. 2A; Movie 1). Our analysis revealed approximately 1140±60 nuclei in hatchlings and approximately 1400±118 in adults, pointing at a significant increase in cell numbers as the animals attained larger sizes (Fig. 2A,B). As we compared adult animals to hatchlings, we observed a notable increase in the area stained by the lipid vital dye BODIPY (Fig. 2C, in green). This led us to hypothesize that this change could be associated with an augmented number of storage cells. Leveraging the nuclei's position within the animal, their small size, and the intense BODIPY staining surrounding these nuclei, we successfully identified and quantified the number of storage cells in both hatchlings and adult tardigrades. While hatchlings contained 72±10 storage cells, the counts for adult *H. exemplaris* showed they bear 281±64 storage cells, indicating a significant rise in storage cell numbers (Fig. 2C,D; Movie 2). Accordingly, we occasionally observed storage cells that appeared to be in the process of division (Fig. S1). Intriguingly, the difference in the total number of cells, and the difference in the total number of storage cells between hatchlings and adults was similar, suggesting that the overall increase in cell number primarily results from storage cell proliferation. Our results show that the number of cells increases during tardigrade somatic growth and suggest that storage cells are the main cell type contributing to this raise.

### Storage cells predominantly proliferate during animal growth

**Fig. 3.**
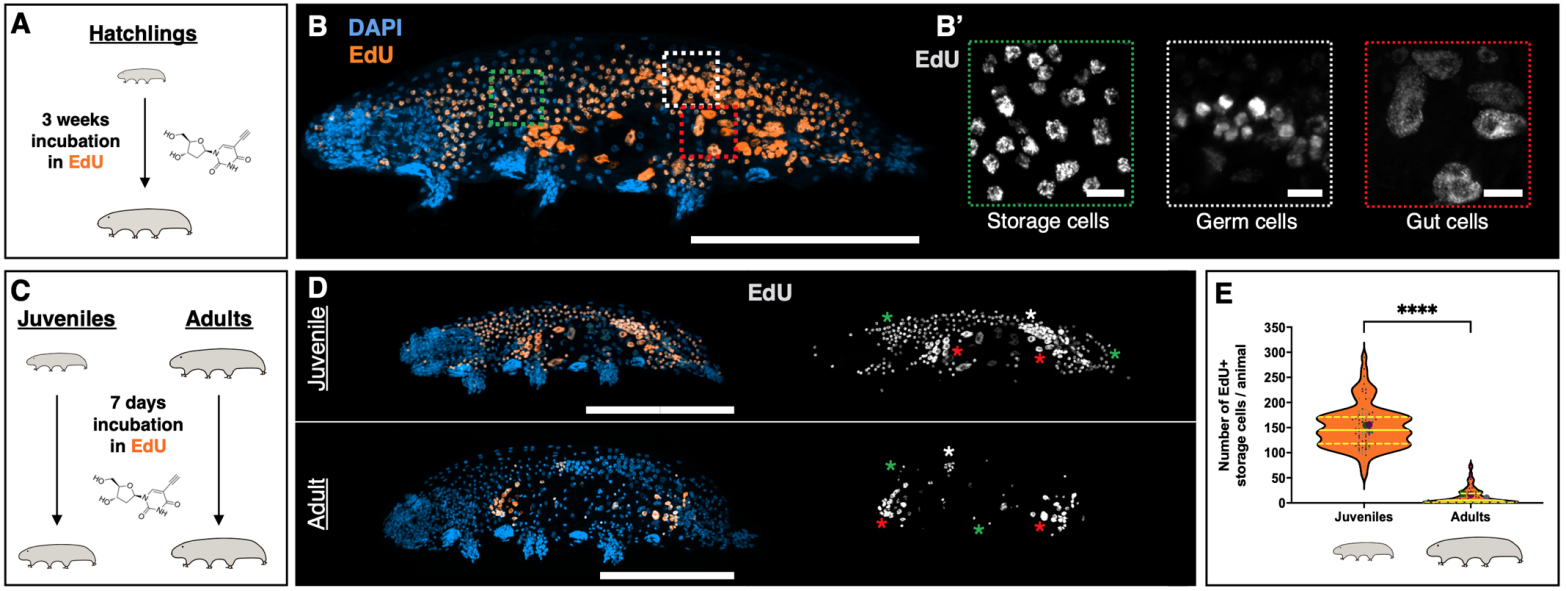
**Storage cell proliferation predominantly occurs during somatic growth.** (A) Schematic depicting the 3-week EdU incubation experiment, concerning B. (B) Representative image of tardigrade upon 3-week exposure to EdU showing nuclei (DAPI, blue) and EdU^+^ cells (orange). (B′) Magnification of regions outlined in B, highlighting the three main replicative cell types labeled with EdU (gray). (C) Experimental schematics for 7-day EdU exposure (D,E). (D) Representative images of juvenile (top) and adult (bottom) after 1-week EdU exposure, displaying nuclei (DAPI, blue) and EdU^+^ cells (orange). Right images show only EdU^+^ cells (gray). (E) Violin plots showing the number of EdU^+^ storage cells in juveniles (*n*=95) and adults (*n*=90). Plotted values belong to three independent experiments. Each quantified animal is represented by a colored dot, with distinct colors corresponding to individual experiments. Larger dots represent each experiment's mean. Plot description as in Fig. 2B. Images shown are maximum projections of the animals' full depth, except for B′, which focuses on specific cell types using sub-stack projections. Asterisks denote EdU^+^ cell types (storage cells, green; germ cells, white; gut cells, red). ****, *P*≤0.0001. Scale bars: 100 µm in B, D; 5 µm in B′.

To investigate cell proliferation at the molecular level, we monitored DNA replication via the incorporation of the thymidine analog EdU, a marker previously employed successfully in tardigrades (Gross et al., 2018). Initially, we collected hatchlings and incubated them in EdU for 3 weeks (Fig. 3A), approximately the duration required for a hatchling to develop into a fully-grown adult when kept at 15°C and fed *ad libitum* (Fig. 1C). This approach had the potential of allowing us to identify any DNA replication that may take place during *H. exemplaris* growth. We detected EdU incorporation in storage cells, germ cells inside the ovary, and gut cells (Fig. 3B-B′). Interestingly, there was an absence of EdU incorporation in any other organ, including epidermal cells, brain cells, ganglia, buccal apparatus, Malpighian tubules, and claw glands (Fig. 3B). While it is known that gut cells incorporate EdU in *H. exemplaris* (Gross et al., 2018), our observation represents the first molecular evidence demonstrating DNA replication, and thus cell proliferation, in tardigrade storage cells.

Tardigrade germ cells reside in the anterior end of the ovary and derive from primordial germ cells that are specified during embryonic development (Heikes et al., 2023). They form germ cell clusters by undergoing mitosis followed by incomplete cytokinesis (Jezierska et al., 2021; Poprawa et al., 2015), thus being expected to incorporate EdU. Within these clusters, the cell completing meiosis develops into an oocyte, while the remaining differentiate into trophocytes (nourishing cells) (Jezierska et al., 2021; Poprawa et al., 2015). Accordingly, in addition to EdU incorporation in germ cells, we observed EdU signal in trophocytes and oocytes of *H. exemplaris* (Fig. S2), corroborating the notion that these cell types are the progeny of germ cell clusters.

Next, we explored whether storage cell proliferation varied between (growing) juveniles and (fully-grown) adults. Juveniles and adults were incubated in EdU for 7 days (Fig. 3C), a duration sufficient for all animals to experience at least one molting event when kept at 15°C. We observed that growing juveniles incorporated EdU in a large number of storage cells, whereas we could only detect a limited number of EdU^+^ storage cells in adults (Fig. 3D, green asterisks), rendering the differences highly significant (Fig. 3E). Juveniles' EdU incorporation in a substantial number of storage cells occurred regardless of the animals' reproductive status at the time of fixation (non-gravid, Fig. 3D; gravid, or laying eggs, Fig. S3). Our findings first show that the majority of storage cell number expansion takes place during tardigrade growth. Second, they evidence that storage cell proliferation also happens, although to a much-lessened extent, after tardigrades have reached their fully-grown status.

### Starvation halts animal growth and suppresses storage cell proliferation

**Fig. 4.**
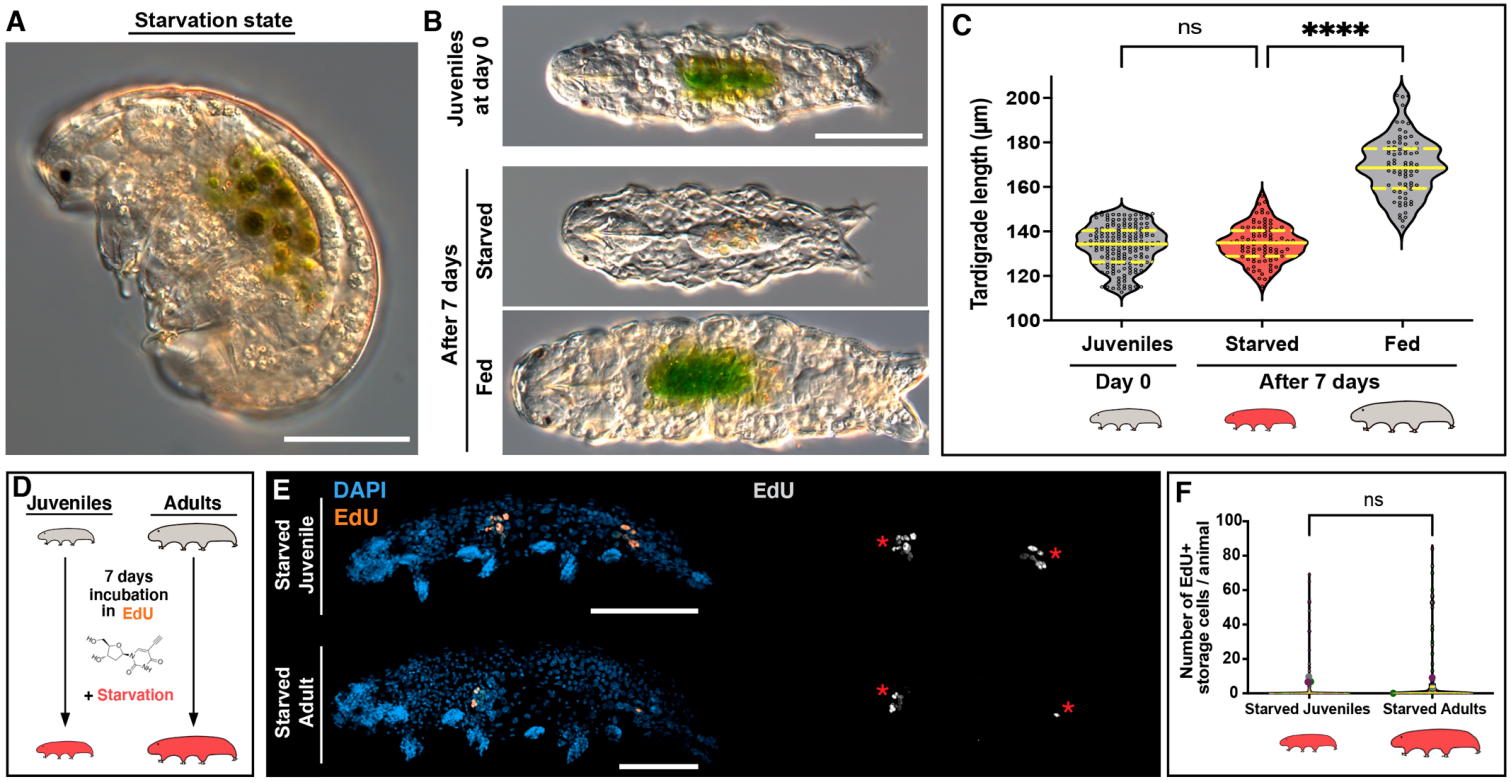
**Impact of starvation on growth and storage cell proliferation.** (A) Representative image of *H. exemplaris* in the described ‘starvation state’. (B) Representative images of *H. exemplaris* juveniles at day 0 and at day 7 under starved and fed conditions. (C) Violin plots illustrating tardigrade length in juveniles at day 0 (*n*=172) and at day 7 under starved (*n*=92) and fed (*n*=80) conditions. Plot description follows Fig. 2B. (D) Experimental schematics for 7-day EdU exposure in starved juveniles and adults (E,F). (E) Representative images of starved juvenile (top) and starved adult (bottom) after 1-week EdU exposure, showing nuclei (DAPI, blue) and EdU^+^ cells (orange). Right images display only EdU^+^ cells (gray). Red asterisks indicate EdU^+^ gut cells. The full depth of the animals was captured in confocal z-stacks, and the images shown are maximum projections. (F) Violin plots showing the number of EdU^+^ storage cells in starved juveniles (*n*=115) and starved adults (*n*=112). Plotted values belong to three independent experiments. Plot conventions are consistent with Fig. 3E. Red tardigrade schematics indicate starved condition. ns=non-significant; ****=*P*≤0.0001. Scale bars: 50 µm.

Tardigrades exhibit remarkable starvation tolerance, enduring several weeks without food (Reuner et al., 2010). Although prior studies noted that starvation reduces storage cell size (Reuner et al., 2010), little is known about its potential effects on tardigrade growth and cell proliferation. To assess this, we first collected small juveniles of *H. exemplaris* and transferred them to dishes with and without *Chlorococcum* algae (‘Fed’ and ‘Starved’ conditions). We observed that the majority of starved animals entered a contracted, resting state (Fig. 4A) shortly after food deprivation. Upon reintroducing algae after 1 week, they resumed activity within minutes, and their guts were filled with algae within 24 h (Fig. S4A), indicating reversibility of the ‘starvation state’. We also noticed a marked reduction in lipid staining inside the storage cells of starved individuals after 1 week (Fig. S4B), suggesting that the fat reserves of storage cells were utilized to sustain the animals during starvation. Additionally, we collected small juveniles again, measured their length, and exposed them to the aforementioned conditions. After 7 days, fed individuals exhibited significant growth, while starved ones showed no substantial increase in size (Fig. 4B,C), implying that starvation induces growth arrest in tardigrades.

To assess cell proliferation during food deprivation, we simultaneously exposed juveniles and adults to EdU and starvation for 7 days (Fig. 4D). In both cases, we found a complete absence of EdU incorporation in storage cells, with rare exceptions where a few storage cells were EdU^+^ (Fig. 4E,F). Essentially, in most individuals of either growth stage we could only detect EdU^+^ gut cells (Fig. 4E, red asterisks). These results align with a previous study, which showed that starved adult *H. exemplaris* incubated with EdU for up to 4 days only exhibited EdU incorporation in gut cells (Gross et al., 2018). Altogether, these findings demonstrate that starvation arrests tardigrade growth and inhibits EdU incorporation in storage cells, unambiguously linking storage cell proliferation to growth.

### EdU incorporation in storage cells takes place during molting

**Fig. 5.**
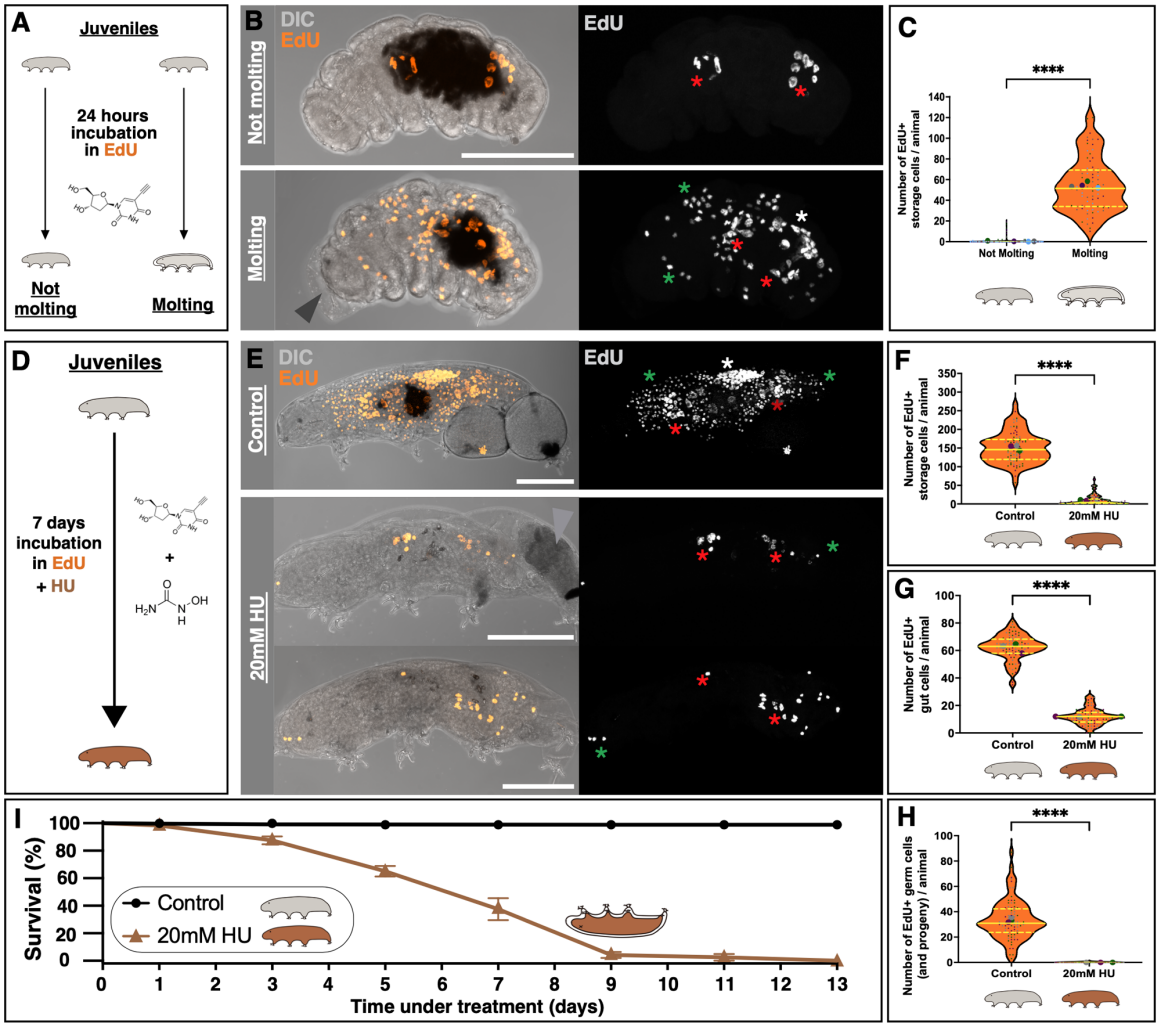
**Storage cells undergo replication during molting, while blocking replication results in molting-related fatalities.** (A) Experimental schematics for 24-h EdU exposure (B,C). (B) Representative images of non-molting (top) and molting (bottom) tardigrades upon 24-h EdU exposure, illustrating animal morphology (DIC, gray) and EdU^+^ cells (orange). Arrowhead indicates the old cuticle being molted. Right images show only EdU^+^ cells (gray). (C) Violin plots displaying the number of EdU^+^ storage cells in non-molting (*n*=452) and molting animals (*n*=76). Plotted values belong to four independent experiments. (D) Schematic illustrating the 7-day experiment exposing tardigrades to EdU and HU simultaneously, concerning E, H. (E) Representative images of control (-HU) and 20 mM HU-treated animals after 1-week EdU exposure, showing animal morphology (DIC, gray) and EdU^+^ cells (orange). Arrowhead indicates a laid egg that has degraded. Right images display only EdU^+^ cells (gray). Maximum projections of the animals' full depth are shown. Asterisks as in Fig. 3. Scale bars: 50 µm. (F-H) Violin plots showing the number of EdU^+^ storage cells (F), gut cells (G), and germ cells (including their progeny: oocytes and trophocytes; H) in control (n=80) and 20 mM HU-treated animals (*n*=93). Plotted values belong to three independent experiments. All plot conventions as in Fig. 3E. ****, *P*≤0.0001. (I) *H. exemplaris* 13-days survival curve in control (-HU) versus 20 mM HU conditions (*n*=120, across three independent experiments). Means±standard errors of the mean (s.e.m.) are plotted. Brown tardigrade schematics indicate 20 mM HU condition. The upside-down brown tardigrade schematic represents animal death during molting.

To investigate whether DNA replication in storage cells occurs at specific stages of *H. exemplaris* post-embryonic development or is a continuous process throughout growth, we conducted 24-h EdU incubations in juvenile animals, including those approaching molting (Fig. 5A). The vast majority of animals that did not molt during the 24-h EdU incubation exhibited no EdU incorporation in storage cells (Fig. 5B,C); only gut cells displayed EdU labeling (red asterisks, Fig. 5B). In contrast, juveniles fixed during molting displayed a significant number of EdU^+^ storage cells (green asterisks in Fig. 5B; Fig. 5C), in addition to EdU^+^ gut and germ cells (red and white asterisks, respectively; Fig. 5B). These results strongly associate storage cell proliferation with the molting process (Fisher's exact test: *P*<0.0001; Table S1). Hence, our findings indicate that storage cell (and germ cell) proliferation does not occur constantly during tardigrade growth but rather in bursts at each ecdysis.

### Hampering DNA replication progression results in tardigrade death during molting

This prompted us to assess the potential relevance of DNA replication for the molting process. To this purpose, we used hydroxyurea (HU), a drug known to effectively slow down the progression of replication forks and, consequently, inhibit EdU incorporation, as demonstrated in other aquatic invertebrates (Quiroga-Artigas et al., 2022; Ramon-Mateu et al., 2019). We employed a 20 mM HU dose in our experiments, strategically positioned between concentrations commonly applied in yeast (∼100 mM) and human cell cultures (1-5 mM) (Ovejero et al., 2022). This decision was based on the anticipation that tardigrade cells, lacking a protective cell wall like yeast, but sheltered by a robust cuticle enveloping the entire organism, would exhibit intermediate sensitivity to HU compared to these reference systems. Additionally, to minimize potential side effects of HU and concentrate on its impact on restricting DNA replication, we opted for a lower HU concentration than those applied in other ecdysozoans (Lozano et al., 2006; Sweeney et al., 2012). First, to ensure that HU successfully reduces EdU incorporation in replicative cells of *H. exemplaris*, we simultaneously exposed juvenile animals to HU and EdU for 7 days (Fig. 5D). We observed an overall decrease in EdU incorporation in animals subjected to 20 mM HU compared to control animals (Fig. 5E). Quantification of the number of EdU^+^ storage cells, gut cells, and germ cells (including their progeny: trophocytes and oocytes) in both control and HU-treated condition revealed a highly significant reduction in all cases for animals under HU treatment (Fig. 5F-H).

Having confirmed that HU disrupts replication progression in *H. exemplaris* by significantly reducing EdU incorporation in all replicative cell types, we then assessed its effects on molting. Intriguingly, in the above experiments, we recurrently observed that by day 7, a substantial number of animals had perished, and we noticed that all deceased individuals had died during the molting process (Fig. 5E). We then incubated *H. exemplaris* specimens in 20 mM HU and monitored mortality for up to 13 days. After 9 days of incubation, allowing sufficient time for all treated animals to molt, we found that 96%±4% of the HU-treated animals had perished (Fig. 5I). Consistent with our initial observations, all fatalities occurred specifically during ecdysis (Fig. S5), irrespective of the day we documented our survival results. Notably, animals that had not yet started molting remained healthy despite being exposed to identical HU conditions. Ecdysis-associated deaths were unrelated to oviposition or egg viability during molting, as evidenced by examples of animals that had laid eggs or not (Fig. 5E; Fig. S5). Altogether, our findings suggest that hindering DNA replication leads to molting-related fatalities.

## DISCUSSION

**Fig. 6.**
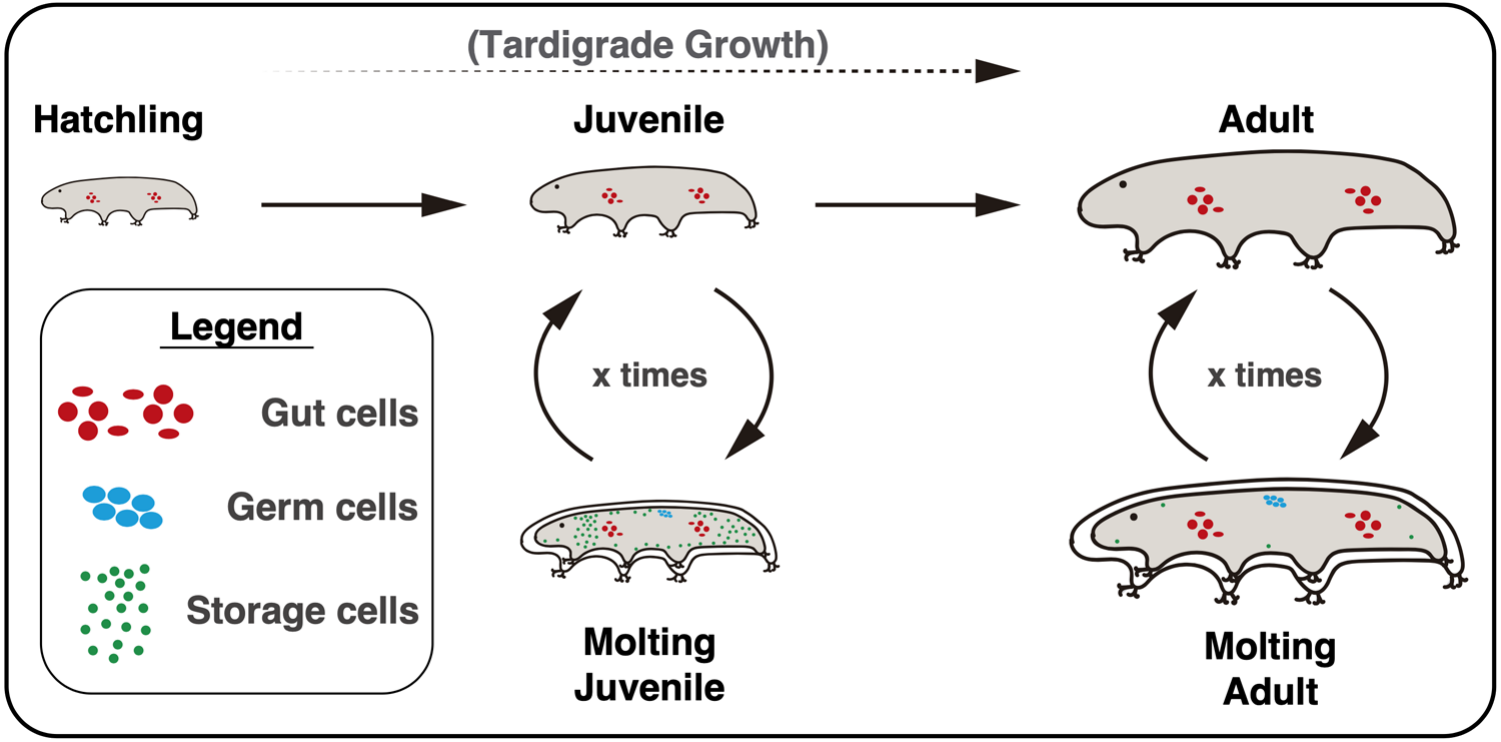
**Overview of DNA replication events in tardigrade post-embryonic development.** Replicative gut cells are represented as red circles and ovals. Replicative germ cells are represented as blue circles. Replicative storage cells are represented as small green circles. In summary, gut cells in the anterior and posterior parts of the midgut replicate continuously, while germ and storage cells only undergo replication at each molting event. The number of replicative storage cells decreases markedly as tardigrades reach their fully-grown adult size, highlighting that most storage cell proliferation occurs during somatic growth.

This study addresses cell proliferation during tardigrade post-embryonic development. Using the emerging model *H. exemplaris*, we illustrate how tardigrade storage cells primarily undergo proliferation during animal growth, leading to an increase in their numbers. We reveal that food deprivation induces a reversible ‘starvation state’ in *H. exemplaris*, during which both growth and storage cell proliferation are halted. Our 24-h EdU pulse experiments establish a clear association between molting and storage cell DNA replication and allowed us to illustrate a comprehensive representation of the DNA replication events occurring during tardigrade post-embryonic life for the three main replicative cell types we identified (Fig. 6). Furthermore, we establish that proper DNA replication is essential for tardigrade survival, as its disruption results in mortality during molting, the sole phase in which gut, germ, and storage cells undergo replication simultaneously (Fig. 6).

*H. exemplaris* does not present a defined number of cells, typical of eutelic animals, as its cell count increases with age and body growth (Fig. 2B). This is reinforced by the observed variability in the total number of cells among individuals of the same species (Fig. 2B). While we have demonstrated that this increase in cell number is primarily attributed to storage cell proliferation (Fig. 2D; Fig. 3), we also detected cell proliferation in the gut and ovary. However, these proliferative events are involved in cell replacement and the formation and sustenance of oocytes, respectively, rather than in overall cell number increase (Gross et al., 2018; Jezierska et al., 2021; Poprawa et al., 2015). In contrast to observations from decades ago in various species of *Macrobiotus*, where some ganglion and claw gland cells were interpreted as mitotic (Bertolani, 1970a), our investigations did not reveal any evidence of cell proliferation in any other organs or tissues of *H. exemplaris* (Fig. 3B; Fig. S2). Thus, storage cells represent the primary cell type in *H. exemplaris* that undergoes proliferation and results in a cell number expansion.

Based on our findings, *H. exemplaris* cannot be categorized as entirely eutelic. Our study, coupled with the observation of mitosis in storage cells of the tardigrade *R. coronifer* (Czernekova and Jönsson, 2016), and the detected variations in organ cell numbers among different tardigrade species (Bertolani, 1970a,b), strongly suggests that the absence of complete eutely may be a widespread characteristic among tardigrades. However, the absence of cell proliferation in specific organs, such as the epidermis and the nervous system, illustrates that some degree of cell constancy may exist in various tardigrade organs. To gain a more comprehensive understanding, it would be valuable to reevaluate the proliferative capacities of different cell types in a range of tardigrade species using contemporary molecular techniques like EdU incorporation. This approach could provide insights into whether the absence of cell proliferation in certain organs is a common feature shared across different tardigrade clades, and whether lack of complete eutely is indeed a general characteristic in the phylum.

The presence of cell proliferation in a limited number of cell types implies that the growth of most tardigrade organs primarily occurs through an increase in cell size rather than through cell proliferation. In this context, an increment in the number of storage cells may serve as a mechanism to support a larger body size and the associated higher demand for stored materials. This hypothesis has been previously suggested (Czernekova and Jönsson, 2016), and our data offer two pieces of evidence to support it. First, we show that growth arrest induced by starvation in juvenile tardigrades leads to the inhibition of storage cell proliferation (Fig. 4). Second, in adult *H. exemplaris*, where full growth has been achieved, the majority of storage cells within the animal no longer undergo proliferation (Fig. 3D,E). From another perspective, considering that storage cells also contribute to vitellogenesis (Hyra et al., 2016; Poprawa, 2006), and given that a larger body size allows for a greater number of eggs to develop in the ovary, we postulate that an increase in storage cell numbers throughout tardigrade growth could further enhance the capacity to produce and nourish a larger number of eggs per individual. This aligns with our unreported observations that adult animals consistently produce more eggs than juveniles.

While anterior and posterior midgut cells maintain a constant rate of proliferation (Fig. 6; Gross et al., 2018), germ and storage cells appear to be cell cycle-arrested, presumably in G_1_ or in G_o_, only resuming cycling to undergo DNA replication during molting (Fig. 5B,C; Fig. 6). Correspondingly, a similar association between storage cell mitotic activity and molting has been reported in the tardigrade species *R. coronifer* (Czernekova and Jönsson, 2016). These observations would imply that a specific cue, received at the time of molting, prompts these cell types to resume the cell cycle towards DNA replication. What could be the nature of such a signal? Tardigrades share orthologs of several genes associated with arthropod molting processes, including the ecdysone receptor and early activated genes driven by ecdysone (Schumann et al., 2018). This indicates a certain level of conservation of molting determinants within the Panarthropoda (de Oliveira et al., 2019; Schumann et al., 2018). Notably, recent research has shown that low ecdysone levels can stimulate cell proliferation in *Drosophila* wing imaginal discs (Perez-Mockus et al., 2023). Downstream of ecdysteroid hormones, a cascade of evolutionarily ancient ecdysis-related neuropeptides (ERNs) plays crucial roles during ecdysis (de Oliveira et al., 2019). Originally responsible for regulating life cycle transitions in both molting and non-molting phyla, these ERNs have been co-opted to orchestrate the molting process in ecdysozoans (de Oliveira et al., 2019; Zieger et al., 2021). Some of the genes encoding these ERNs are conserved in tardigrades, including eclosion hormone (EH) and crustacean cardioactive neuropeptide (CCAP), which are upregulated at hatching (Zieger et al., 2021). Intriguingly, *H. exemplaris* presents five EH and two CCAP paralogs (Koziol, 2018; Zieger et al., 2021). Along these lines, a neuropeptide-receptor couple has been found to induce meiotic progression in jellyfish oocytes arrested at prophase I (Quiroga-Artigas et al., 2020), suggesting that ERN-receptor couples could also regulate cell cycle progression. Collectively, we propose that ecdysteroid-type molting hormones and/or ERNs may serve as triggers for the resumption of the germ and storage cell cycle in tardigrades.

The question emerges as to why HU kills *H. exemplaris* during molting. Our primary intent behind using HU was to inhibit the production of deoxyribonucleotides, thereby restricting DNA replication. Tardigrade molting events are likely the most energy-demanding stages of their post-embryonic development, since they involve replacing the buccal apparatus, claws, and the entire cuticle (Czerneková and Vinopal, 2021), synchronization with egg laying (Altiero et al., 2018), and bursts of storage and germ cell proliferation (Fig. 5B,C). Given that gut cells undergo permanent renewal (Gross et al., 2018), animals' death during molting could reflect a profound impact of disrupting gut cell proliferation, consequently affecting overall energy absorption. In this scenario, the decrease in gut cell proliferation would have a more deleterious effect on survival than that of storage cells.

However, additional effects of HU may impact replicative cells directly or have a systemic effect. Firstly, HU induces oxidative stress, causing DNA damage (Davies et al., 2009) and leading to cytotoxic effects, such as membrane damage and mitochondrial defects (Singh and Xu, 2016). Secondly, associated with replication but not directly linked to the absence of DNA synthesis, HU's effect on slowing down replication fork progression triggers a response inhibiting the firing of late origins (Kliewer and Schulman, 1998). Consequently, the coordination of the replicative program with other DNA-related processes, such as transcription, is perturbed (Hoffman et al., 2015). As shown in other ecdysozoans, ecdysis is a highly transcriptionally active process (Schumann et al., 2018). Moreover, recent research has demonstrated that successful molting in the nematode *Caenorhabditis elegans* requires rhythmic accumulation of transcription factors, which, in turn, relies on rhythmic transcription (Meeuse et al., 2023). Therefore, HU treatment could enhance tardigrade lethality during molting due to profound alterations in the execution of the ecdysis transcriptional schedule. Lastly, HU also leads to DNA break accumulation when replication fork stalling is long-lasting (Singh and Xu, 2016), triggering a robust DNA damage response (Ciccia and Elledge, 2010). In the context of *H. exemplaris*, where there is no storage cell proliferation between molting cycles, DNA damage signals may be modest or remain undetected. However, they may translate into a systemic death signal when this insult occurs during a burst of storage cell proliferation. In agreement, DNA damage in proliferative cell types has recently been shown to trigger an alert in the whole animal that accelerates inflammatory and aging outputs (Matos-Rodrigues et al., 2023).

In view of our results demonstrating that HU significantly reduces EdU incorporation in all three replicative cell types (Fig. 5F-H), the consistent coincidence of HU-induced animal death with the molting period (Fig. 5E; Fig. S5), and the simultaneous replication of all replicative cell types during ecdysis (Fig. 5B, Fig. 6), we lean towards the interpretation that HU-induced molting-related fatalities are closely linked to the drug's impact on DNA replication-related metabolism.

In summary, our study provides the first molecular evidence of storage cell proliferation in a tardigrade species. It determines that tardigrades do not present cell constancy during post-embryonic development, definitively establishing tardigrades as non-eutelic animals. Moreover, our data shed new light on tardigrade cell biology and establish a clear connection between tardigrade ecdysis and cell cycle transitions. The finding that storage cells undergo replication opens new possibilities for *in vitro* culture of this cell type and suggests its potential as a valuable model for studying cell cycle dynamics, responses to replication stress, and DNA damage control in tardigrades. With the advent of transgenesis (Tanaka et al., 2023) and CRISPR (Kumagai et al., 2022) technologies in tardigrades, along with the established RNAi methods (Boothby et al., 2017; Tenlen et al., 2013), *H. exemplaris* is increasingly becoming a promising genetic experimental model, altogether marking an exciting era in tardigrade research.

## MATERIALS AND METHODS

### Animal husbandry and drug treatment

*H. exemplaris* (Z151 strain) husbandry was conducted as previously described (Roszkowska et al., 2021), with minor modifications. In brief, tardigrades were maintained in an incubator at 15°C, placed inside 55 mm diameter plastic Petri dishes filled halfway with spring water (Volvic) filtered through a 0.2 µm mesh. To facilitate tardigrade movement, the bottom of the dishes was scratched with sandpaper. Photoperiod and relative humidity inside the incubator were not monitored. Animals were fed *ad libitum* with *Chlorococcum* algae, cultivated in 50 ml tubes with BG-11 Growth Media (Gibco, A1379901). Water changes were performed every 2 weeks. All experimental incubations lasting ≥24 h were carried out under these same conditions in 35 mm diameter dishes, placed inside cardboard boxes to avoid degradation of potentially photosensitive compounds.

Tardigrades were subjected to incubation with the ribonucleotide reductase inhibitor hydroxyurea (HU; Sigma-Aldrich, H8627) at a concentration of 20 mM, dissolved in filtered spring water (FSW) for the duration of the experiment. Renewal of HU took place every 2-3 days to ensure its continued effectiveness.

### DAPI and BODIPY staining

Animals were collected and filtered through a 40 µm mesh, then transferred to a glass depression slide within a humid chamber using a glass Pasteur pipette. Whenever possible, samples were kept in darkness for the duration of the experiment. To label neutral lipids, live specimens were incubated in 10 µg/ml BODIPY (Difluoro{2-[1-(3,5-dimethyl-2H-pyrrol-2-ylidene-N)ethyl]-3,5-dimethyl-1H-pyrrolato-N}boron; Sigma-Aldrich, 790389) diluted in FSW for 30 min. Subsequently, samples were rinsed three times with FSW and fixed in 4% PFA in 1x PBS for 1 h at room temperature (RT). After fixation, three 15-min washes in 1x PBS were performed. Samples were then incubated in the DNA fluorescent stain DAPI (4′,6-diamidino-2-phenylindole; Sigma-Aldrich, D9542) at a concentration of 1 µg/ml in 1x PBS for 30 min. Following this step, the samples were washed three times in 1x PBS for 15 min, transferred to a slide, and mounted in ProLong Gold Antifade Mountant (Invitrogen, P36930) prior to confocal imaging.

### EdU experiments

To detect DNA replication, animals were incubated in 100 µM EdU (EdU Click-iT™ Cell Proliferation Kit for Imaging, Alexa Fluor 488 Dye; Invitrogen, C10337) diluted in FSW containing algae (except during experimental starvation) for specific durations (24 h, 7 days, or 3 weeks). In the 3-week experiment, EdU was refreshed weekly. Following EdU exposure, tardigrades were collected, transferred to a glass depression slide, and fixed with 4% PFA in 1x PBS+1% Triton X-100 (PTx) for 1 h at RT. After fixation, the samples underwent three 15-min washes in PTx. Subsequently, they were blocked for 2 h in a 0.2 µm-filtered blocking solution containing 10% bovine serum albumin (BSA) in 1x PBS. The Click-iT EdU detection reaction was carried out for 1 h at RT according to the manufacturer's instructions. After the detection reaction, four 10-min PTx washes were conducted. DAPI was used to stain nuclei, and samples were mounted in ProLong Gold for confocal imaging, as described above. In some cases, tardigrades were euthanized with 10% EtOH before fixation to prevent the animals from contracting during exposure to PFA.

### Imaging, length measurements, cell counting and statistics

To capture live images of tardigrades, animals were anesthetized using 20 mM levamisole hydrochloride (MedChemExpress, HY-13666) in mqH_2_O and mounted on slides, covered with coverslips. Clay was positioned in each corner of the coverslip to prevent compression, except for the intentionally compressed specimen in Fig. 1B. Differential interference contrast (DIC) color images were taken with a Leica K3C digital color camera attached to a compound light microscope (Leica Thunder). For measuring the body length of tardigrades, measurements were conducted using ImageJ (Schneider et al., 2012). The measurement was taken from the head to the juncture on the posterior-most segment with legs (Vasanthan and Stone, 2020). For starved tardigrades, measurements were obtained immediately after animals came back to an active state, ensuring their full body extension.

Fluorescently labeled images were acquired using a Zeiss LSM980 confocal microscope, equipped with an Airyscan2 module. To ensure consistency when comparing samples within a given experiment, identical scanning parameters were applied to all conditions in each independent experiment. Confocal Z-stacks and projections were processed and adjusted for brightness and contrast in ImageJ, using K. Terretaz's visualization toolset (https://github.com/kwolbachia/Visualization_toolset).

To count the total number of cells in hatchlings and adults, Imaris software (Oxford instrument) was employed. Nuclei were highlighted using custom thresholding to allow for quantification. Quantifications of BODIPY-stained storage cells and EdU^+^ cells were performed manually in ImageJ. In all instances, z-stacks encompassing the entire depth of the animals were utilized. Movies 1 and 2 were assembled using Imaris and ImageJ software, respectively. Tardigrade schematics and figure compilation were generated using Adobe Illustrator.

To determine statistical significance, normality of all datasets was first assessed using the Shapiro-Wilk test. Two-tailed Student's *t*-test was conducted for the dataset concerning total number of storage cells (Fig. 2D). For all other two-way comparisons, Mann–Whitney *U* nonparametric tests were employed. The Kruskal–Wallis test was used for statistical analyses in Fig. 4C. Fisher's exact test was selected for the association assessment, based on a 2×2 contingency table. Statistical significance for all quantitative comparisons is represented as ****, where *P*-value≤0.0001. Graphs and statistical analyses were performed using GraphPad Prism 9.

## Supplementary Material

10.1242/biolopen.060299_sup1 Supplementary information
